# Effectiveness of Autologous Plasma Rich in Growth Factors on Healing of Extraction Socket—A Systematic Review

**DOI:** 10.3390/jcm15020593

**Published:** 2026-01-12

**Authors:** Yasser Eid Al-Thobaiti, Yousef Al Thomali, Sakeenabi Basha, Roshan Noor Mohamed, Azzah O. Alhazmi, Thamer E. Alzahrani, Mohammed Khalil Fahmi, Ali Alqarni

**Affiliations:** 1 Department of Oral and Maxillofacial Surgery and Diagnostic Sciences, Faculty of Dentistry, Taif University, Taif 21944, Saudi Arabia; 2 Department of Preventive Dentistry, Faculty of Dentistry, Taif University, Taif 21944, Saudi Arabia; 3 Department of Restorative Dentistry, Faculty of Dentistry, Taif University, Taif 21944, Saudi Arabia

**Keywords:** plasma rich in growth factors, soft tissue healing, tooth extraction, post-operative pain

## Abstract

**Objectives**: This systematic review aims to evaluate the effectiveness of autologous Plasma Rich in Growth Factors (PRGF) in enhancing post-extraction socket healing by synthesizing evidence from randomized controlled trials and assessing outcomes related to bone regeneration, soft-tissue healing, and postoperative discomfort. **Methods**: A comprehensive search was conducted in MEDLINE, Embase, Cochrane Library, Scopus, Web of Science, and CINAHL, using a fully reproducible Boolean search strategy. Non-English studies were screened but excluded only when a reliable translation was not feasible. Only randomized controlled trials (RCTs) involving PRGF application in human extraction sockets were included. Risk of bias was assessed using the Cochrane RoB2 tool. A meta-analysis could not be performed due to substantial heterogeneity in PRGF preparation protocols, follow-up duration, and outcome measurements. **Results**: Seven RCTs met the eligibility criteria. Four studies demonstrated significantly improved healing outcomes in the PRGF group compared with controls, whereas two studies reported comparable results. Pain reduction was consistently observed in PRGF-treated sockets in studies that reported standardized postoperative analgesic protocols. Mineralized tissue formation favored PRGF in high-quality trials. Considerable heterogeneity was identified in PRGF centrifugation parameters, outcome tools, and evaluation timelines. **Conclusions**: Evidence from current RCTs supports PRGF as an effective and well-established adjunct for enhancing early post-extraction healing. The novelty of this review lies in its updated methodological rigor, corrected risk-of-bias analysis, standardized data handling, and clarification of long-standing gaps in reporting PRGF preparation variability. Future trials with standardized PRGF protocols and long-term follow-up are needed to improve comparability and strengthen clinical recommendations.

## 1. Introduction

Tooth extraction is a prevalent dental procedure globally. Despite the straightforward nature of the extraction process, post-extraction complications, including delayed healing, trismus, pain, infections, and alveolar osteitis, pose significant challenges for clinicians and patients [1,2]. Extraction socket healing involves a series of biological events, such as blood clot formation, angiogenesis, and bone regeneration [1,2,3]. These processes may be impaired or delayed by factors including inadequate blood supply, systemic health conditions, or extraction-related trauma [4,5]. Consequently, there is increasing interest in advanced regenerative therapies to improve healing outcomes and reduce patient morbidity [6,7,8].

Plasma Rich in Growth Factors (PRGF), an autologous platelet-derived concentrate, represents a promising therapeutic modality in regenerative medicine and dentistry [9,10,11,12,13,14,15,16,17,18,19,20,21,22,23]. PRGF is derived from the patient’s own blood and contains high concentrations of biologically active growth factors, including platelet-derived growth factor (PDGF), transforming growth factor-beta (TGF-β), and vascular endothelial growth factor (VEGF), which are critical for tissue regeneration and repair [10]. These growth factors facilitate cell proliferation, angiogenesis, and osteogenesis, thereby supporting both soft tissue and bone regeneration after tooth extraction [10,11,12,13,14,15,16,17,18,19,20].

Numerous studies have highlighted PRGF’s potential to accelerate postextraction healing while reducing inflammation and associated pain [9,10,11,12,13,14,15,16,17,18,19,20,21,22,23]. For instance, randomized clinical trials have demonstrated that PRGF enhances socket healing by improving early epithelialization and bone density compared to conventional blood clot healing [20,23]. In addition to its regenerative benefits, PRGF exhibits anti-inflammatory and antimicrobial properties, which can further reduce postoperative complications such as pain and infection [19,20,21,22,23,24,25,26,27,28].

The use of PRGF also holds significant advantages over other biomaterials, such as platelet-rich plasma (PRP) or alloplastic grafts, as it is 100% autologous, biocompatible, and eliminates the risk of immunological reactions or disease transmission. Moreover, its ease of preparation and cost-effectiveness make it a practical option for widespread clinical use in dental extractions [15,20].

Despite long-standing clinical applications, existing studies reporting PRGF efficacy in extraction socket healing exhibit substantial variability. Differences in centrifugation parameters, platelet yield, application protocols, follow-up periods, and outcome assessment tools have contributed to inconsistent findings. Previous systematic reviews have included heterogeneous platelet-based materials (e.g., PRF, PRP), complicating the interpretation of PRGF-specific benefits.

The originality of the present systematic review lies in its exclusive focus on RCTs evaluating PRGF alone without pooling platelet-rich fibrin or other platelet concentrates, the use of the appropriate Cochrane RoB2 tool, improved search reproducibility, and explicit acknowledgment of PRGF preparation variability. Given these considerations, this systematic review aims to synthesize the best available evidence to evaluate the effectiveness of autologous Plasma Rich in Growth Factors (PRGF) in enhancing post-extraction socket healing by synthesizing evidence from randomized controlled trials and assessing outcomes related to bone regeneration, soft-tissue healing, and postoperative discomfort. This review also attempts to identify the methodological gaps that warrant improved standardization in future research.

## 2. Materials and Methods

The present systematic review was planned, conducted, and reported as per the PRISMA standards of quality for reporting systematic reviews and meta-analyses [29] with registration number CRD42024616707 (Supplementary File S3. PRISMA Main Checklist, Supplementary File S4. PRISMA Abstract Checklist). The approval from the Institutional Review Board was not required.

### 2.1. Questions

The area of focus was to examine the healing of the extraction socket following treatment with Plasma Rich in Growth Factors (PRGF). The research question was defined according to the PICO format as follows:

P (Population/Patients): Original studies in human subjects with post-extraction (Randomized controlled trials and comparative studies with controls or comparable treatment data).

I (Intervention): Autologous PRGF treatment following the extraction of human teeth.

C (comparison): Post extraction socket healed without PRGF.

O (Outcome): Mineralized bone density, non-mineralized tissue healing, pain, swelling, and soft tissue healing in both the Intervention and control groups.

### 2.2. Study Eligibility

Articles published in English or with an available professional translation that evaluated the effectiveness of plasma rich in growth factors (PRGF) in extraction socket healing using control groups were included. Non-English studies were screened; however, studies for which no accurate scientific translation could be assured were excluded with justification due to the risk of misinterpretation of methods or outcomes. Editorial letters, case reports, in vitro experiments, and studies unrelated to the assessment of PRGF effectiveness in extraction socket healing were excluded.

### 2.3. Study Identification

A comprehensive and systematic search of the literature was conducted in accordance with PRISMA 2020 guidelines to identify studies evaluating the effectiveness of Plasma Rich in Growth Factors (PRGF) on extraction socket healing. The electronic databases searched included PubMed (MEDLINE), OVID MEDLINE, Embase, Cochrane Library (Trials and Reviews), SCOPUS, Web of Science, CINAHL, PsycINFO, and ERIC. The search covered all literature published up to February 2025. Search strategies incorporated controlled vocabulary (MeSH, Emtree, CINAHL Headings) and free-text keywords combined using Boolean operators (Supplementary File S2. Search Strategies).

Additionally, reference lists of eligible studies were screened for additional RCTs. The following fully reproducible search strategy was used in MEDLINE and adapted for other databases: (“plasma rich in growth factors” OR PRGF OR “growth factor rich plasma”) AND (“tooth extraction” OR “extraction socket” OR “post-extraction” OR exodontia) AND (“healing” OR “bone regeneration” OR “socket preservation” OR “soft tissue healing”) AND (randomized controlled trial OR RCT). 

### 2.4. Study Selection

All search results were screened independently by two reviewers in two phases: “Title and abstract screening” and “Full-text assessment”. Disagreements were resolved by discussion. Inter-rater reliability was strong (κ = 0.85). Excluded studies were those that did not meet eligibility criteria, including non-randomized designs, use of PRGF in conjunction with additional biomaterials, or inadequate data reporting.

### 2.5. Risk of Bias Assessment

Risk of bias was reassessed using the Cochrane Risk of Bias tool for randomized trials, version 2 (RoB2) [30]. Domains evaluated: Randomization process, Deviations from intended interventions, Missing outcome data, Measurement of outcomes, Selection of reported results. Each domain is graded as a low risk of bias, Moderate risk of bias, serious risk of bias, Critical risk of bias, or no information. Two assessors independently scored each study; disagreements were resolved through consensus (Interrater reliability—0.90).

### 2.6. Data Extraction and Data Synthesis

Data extracted included the following: Two reviewers extracted the data independently using a data extraction sheet. The discrepancies between the reviewers were resolved by consensus through discussion. The following data were extracted from each included study: first author, year of publication, type of study, study quality, sample size, inclusion criteria, treatment type, mean/median change in bone density, measurement criteria, pain measurement, statistical analysis used, and the conclusions by authors. Due to the limited number of included studies (n < 10) and significant clinical heterogeneity, a formal assessment of publication bias using a funnel plot was not performed, as recommended by the Cochrane Handbook, because such analyses lack the power to distinguish chance from true asymmetry (Cochrane Handbook for Systematic Reviews of Interventions, Version 6.3, 2022). Due to significant clinical and methodological heterogeneity among the included studies regarding PRGF preparation protocols, outcome measures, and follow-up periods, a meta-analysis was deemed inappropriate.

## 3. Results

**Figure 1:**
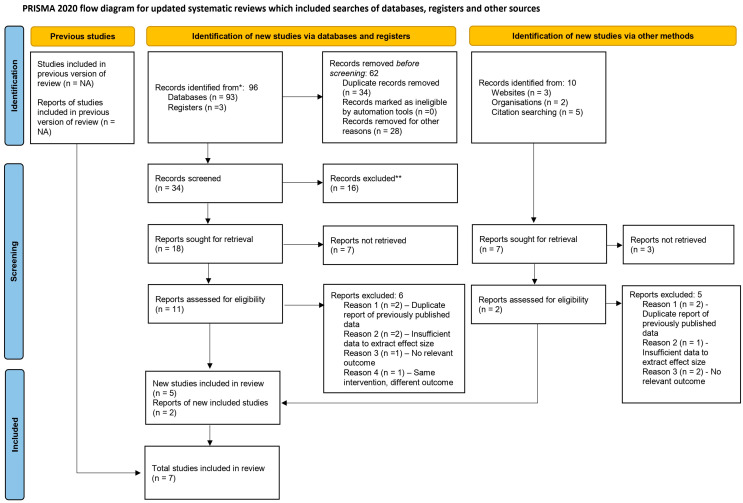
Prisma Flow diagram of studies included in the systematic review. *—studies identified from databases and registers, **—Studies excluded due to duplication.

The study selection process is illustrated in Figure 1. A total of seven randomized controlled trials were included in this review. The list of excluded studies [31,32,33,34,35,36,37,38,39,40,41,42,43,44,45,46,47,48,49,50,51,52,53,54,55,56,57,58,59,60,61,62,63,64,65,66], along with the reasons for exclusion, is provided in Table 1.

Table 2 summarizes the characteristics of the included studies, including study design, participant details, interventions, outcomes, and main conclusions. All seven studies were randomized controlled trials. The total number of participants ranged from 28 to 70, and the follow-up duration varied from 7 days to 12 weeks. Among the seven studies, four reported that PRGF significantly improved post-extraction socket healing compared with the control groups, while two studies found no significant difference between the groups.

Table 3 shows the mean change in mineralized and non-mineralized tissue in the study and control groups and the healing of soft tissues. Among the studies included in the systematic review, Mozzati M. et al. [18] and Brazdeikytė V. et al. showed a significant difference in visual analog scale (VAS) between the PRGF group and control group, with 0.19 cm VAS in the PRGF group and 0.49 cm VAS in the control group in the study by Mozzati M. et al. and 4.4 ± 2.6 VAS in the PRGF group and 5.1 ± 2.8 in the control group in the study by Brazdeikytė V. et al. The study by Anitua E et al. showed a 450.0 ± 106.7 change in mineralized tissue in the PRGF group and 318.2 ± 113.0 in the control group (*p* = 0.0001). Stumbras A et al. showed a 75.5 ± 16.3% change in mineralized tissue among the PRGF group and 46.5 ± 15.2% in the control group (*p* < 0.05).

Table 4 shows the risk of bias assessment. Using the Cochrane RoB 2.0 tool, the assessment revealed 5 studies with low overall risk of bias and 2 with moderate risk of bias. Specific domain ratings for each study are displayed in Table 4. The two studies rated as moderate risk and had concerns were Mozzati et al. [18]—selection bias (allocation concealment not described) and missing data issues; Brazdeikytė et al. [24]—deviations from protocol and outcome measurement bias.

## 4. Discussion

The systematic review evaluated the efficacy of Plasma Rich in Growth Factors (PRGF) in enhancing post-extraction socket healing and reducing postoperative complications such as pain and inflammation. The findings from the included studies [18,22,24,27,28] demonstrate that PRGF has significant potential in improving healing outcomes, although some heterogeneity in results warrants careful interpretation.

Healing Outcomes and Bone Regeneration: The majority of studies [18,22,24,27,28] included in this review reported that PRGF significantly improved bone regeneration and soft tissue healing compared to control groups. For instance, Anitua et al. [28] observed a substantial increase in mineralized tissue density (450.0 ± 106.7) in the PRGF group compared to the control group (318.2 ± 113.0, *p* = 0.0001). Similarly, Stumbras et al. [22] found that PRGF was equally effective as xenografts/allografts in ridge preservation and bone regeneration, with 75.5 ± 16.3% mineralized tissue change in the PRGF group versus 46.5 ± 15.2% in the control group (*p* < 0.05). These findings align with previous research demonstrating that PRGF enhances osteogenesis and angiogenesis, promoting faster and more robust bone regeneration [18,22,24,27,28]. However, Farina et al. [12] reported no significant enhancement in early bone deposition with PRGF compared to controls. This discrepancy may be attributed to differences in study design, such as the use of histomorphometric markers (e.g., CD68+, OCN) that may not capture early-phase bone regeneration as effectively as radiographic or clinical measures. The heterogeneity in methodologies, including variations in PRGF preparation protocols and outcome measurement tools, likely contributed to these contrasting results [12,19].

Follow-up times for bone healing: A notable limitation across the included RCTs is the relatively short follow-up duration, which ranged from as early as 3 days to a maximum of 12 weeks [12,18,19,22,24,27,28]. While these intervals are adequate for assessing early soft-tissue healing, pain reduction, and initial radiographic changes, they are insufficient to fully characterize the long-term dynamics of bone maturation and remodeling. Physiologically, complete mineralization and stabilization of the extraction socket often require several months, and early radiographic proxies may overestimate or underestimate true regenerative potential. Consequently, the positive effects of PRGF documented in the included studies [18,22,24,27,28] should be interpreted as benefits primarily related to early-phase healing rather than definitive evidence of long-term bone regeneration. The absence of extended follow-up also limits the ability to evaluate socket volume stability, late complications, or the sustained quality of regenerated bone. Future RCTs with standardized PRGF protocols and follow-up periods extending beyond 6–12 months are essential to determine whether the early improvements observed translate into durable and clinically meaningful long-term outcomes.

Pain and Inflammation Reduction: PRGF demonstrated consistent efficacy in reducing postoperative pain and inflammation. Mozzati et al. [18] reported significantly lower pain scores in the PRGF group (0.19 cm on the Visual Analog Scale [VAS]) compared to controls (0.49 cm, *p* < 0.05). Similarly, Brazdeikytė et al. [24] found that PRGF-treated patients experienced less postoperative pain (4.4 ± 2.6 VAS) than those treated with PRF (5.1 ± 2.8 VAS, *p* < 0.05). These results are supported by the anti-inflammatory properties of PRGF, which modulate cytokine production (e.g., TNF-α, IL-6) and reduce tissue inflammation [20]. In contrast, O’Sullivan et al. [19] observed no significant differences in pain scores between PRGF and control groups, although secondary outcomes such as mouth opening and dry socket incidence were comparable. This discrepancy may be due to variations in pain assessment tools or differences in patient populations, such as the inclusion of patients with impacted mandibular third molars, which are associated with higher baseline pain levels.

The interpretation of PRGF effectiveness in the included RCTs must also consider variability in patient selection criteria and the lack of uniform reporting on concomitant medication use. Several studies enrolled systemically healthy adults [19,22,24,28] while others included older patients [12] or those with conditions such as alveolar osteitis [27], creating differences in baseline healing capacity. Medications that may influence wound healing, such as NSAIDs, corticosteroids, antibiotics, or chronic systemic therapies, were inconsistently documented across trials and postoperative analgesic regimens varied considerably [12,18,19,22,24,27,28]. Because many of these agents can modify inflammatory responses, angiogenesis, or bone turnover, their inconsistent use represents a potential confounding factor when comparing PRGF outcomes across studies [12].

Safety Profile and Long-Term Efficacy: This systematic review identifies a significant gap in PRGF safety literature. Across the 7 included RCTs [12,18,19,22,24,27,28], adverse event reporting was minimal and inconsistent. Specifically, no serious adverse events attributable to PRGF were reported in any study. Minor complications (transient swelling, infection) were reported in only 2 studies [12,19] and were comparable between the PRGF and control groups. However, the limited long-term follow-up (maximum 12 weeks) in included studies is inadequate for a comprehensive safety assessment. Delayed infections, persistent alveolar osteitis, or delayed healing complications require longer observation. No study systematically evaluated safety outcomes [12,18,19,22,24,27,28]. Future research should include structured adverse event monitoring protocols with standardized definitions; long-term follow-up of ≥6 months; systematic documentation of all complications and safety events; and assessment of PRGF use in medically compromised patients (immunosuppressed, diabetic, smokers) with compromised healing capacity.

Methodological Heterogeneity and Limitations: The studies included in this review [12,18,19,22,24,27,28] exhibited considerable methodological heterogeneity, particularly in PRGF preparation protocols, outcome measures, and follow-up durations. For example, Anitua et al. [28] used a standardized PRGF preparation method, while other studies employed variations in centrifugation speeds and platelet concentrations, which may influence growth factor release and efficacy [15].

An important consideration when interpreting the results of this review is the variability in the clinical conditions under which the included tooth extractions were performed. The RCTs assessed involved diverse extraction scenarios, such as impacted mandibular third molar removal [18,19], routine mandibular molar extraction [24,28], alveolar ridge preservation procedures [22], and the management of alveolar osteitis [27]. These clinical situations differ substantially in baseline inflammation, surgical complexity, extent of tissue trauma, and natural healing capacity. For instance, third molar surgeries typically induce greater postoperative inflammation compared with simple extractions [18,19], while alveolar osteitis presents with an impaired healing environment and delayed epithelialization [27]. Such differences may partly explain the heterogeneity in reported outcomes across studies. Consequently, variations in clinical indication and extraction difficulty should be considered when evaluating PRGF effectiveness, and future trials would benefit from standardized reporting of extraction type, surgical difficulty, and baseline socket conditions (Supplementary Table S1. Heterogenicity assessment).

Additionally, the use of different imaging modalities (e.g., CBCT, histomorphometry) and pain assessment tools (e.g., VAS, NRS) introduced variability in outcome reporting. Owing to the small number of included studies (n = 7), funnel plot evaluation was not meaningful. Meta-analysis was not feasible due to substantial heterogeneity in PRGF preparation methods, outcome assessment tools, and follow-up durations.

Despite these limitations, the overall risk of bias was low [12,19,22,27,28] to moderate [18,24] across studies, with most demonstrating robust randomization and blinding procedures [67]. However, the lack of long-term follow-up in several studies limits the ability to assess the durability of PRGF’s effects on bone regeneration and socket healing.

Comparison with Previous Research: The findings of this review are consistent with prior studies [20,21,22,23,24,25,26], highlighting the regenerative potential of PRGF in dentistry. For instance, Sammartino et al. [21] reported accelerated epithelialization and bone density in PRGF-treated sockets, corroborating the results of Anitua et al. [28] and Stumbras et al. [22]. However, contrasting findings, such as those reported by Farina et al. [12], underscore the need for standardized protocols and larger, multicenter trials to validate PRGF’s efficacy.

## 5. Conclusions

This systematic review synthesizes current evidence from RCTs reinforcing the role of PRGF as a beneficial adjunct in enhancing post-extraction socket healing, reducing pain, and minimizing inflammation. However, methodological heterogeneity and inconsistent findings in some studies highlight the need for standardized protocols and further research. PRGF represents a promising, cost-effective, and biocompatible option for improving post-extraction outcomes, with potential for widespread clinical adoption.

## Figures and Tables

**Table 1:** Excluded studies with reason.

Study	Reason for Exclusion
Fok & Jin (2024) [31]	Review article—not original research/not RCT
Laforgia et al. (2024) [32]	Systematic review—secondary evidence
Al-Maawi et al. (2021) [33]	Systematic review—not primary RCT data
Ahmed et al. (2019) [34]	PRF used; not PRGF
Alzahrani et al. (2017) [35]	PRF used; not PRGF
Anitua et al. (2004) [36]	Narrative description; not clinical study
Anitua et al. (2007) [37]	Not extraction study; PRGF conceptual, not used for extraction socket healing
Anwandter et al. (2016) [38]	PRF (L-PRF) used; not PRGF
Asutay et al. (2017) [39]	PRF used, not PRGF
Borsani et al. (2018) [40]	In vitro study; not human clinical trial
Castro et al. (2021) [41]	PRF variants; not PRGF
Choukroun et al. (2006) [42]	PRF method; not PRGF
Clark et al. (2018) [43]	Mixed interventions; not PRGF-only
Dohan Ehrenfest et al. (2010) [44]	In vitro cell culture; not human clinical study
Dohan Ehrenfest et al. (2012) [45]	In vitro comparative analysis
Dutta et al. (2016) [46]	Comparison included PRP/PRF/HA; not PRGF-specific
Fang et al. (2022) [47]	PRGF not used; CGF fibrin
Girish Kumar et al. (2018) [48]	PRF used; not PRGF
Ghanaati et al. (2018) [49]	Review article
Giudice et al. (2019) [50]	Not PRGF; APRF & LPRF used
Guo et al. (2022) [51]	Animal study (rats)
Hauser et al. (2013) [52]	PRF used; not PRGF
Hatakeyama et al. (2014) [53]	Animal study (dogs)
Kamal et al. (2020) [54]	Condition not relevant (alveolar osteitis)
Kobayashi et al. (2016) [55]	In vitro growth factor release study
Kubesch et al. (2018) [56]	Animal study (in vivo experimental)
Mogharehabed et al. (2014) [57]	Animal study (dogs)
Mozzati et al. (2022) [58]	CGF used; not PRGF
Santos Pereira et al. (2023) [59]	Systematic review—secondary evidence
Schär et al. (2015) [60]	In vitro study
Semkin et al. (2022) [61]	Non-English publication (Russian)
Simon et al. (2009) [62]	Comparative study with bone grafts; not PRGF-only
Srinivas et al. (2018) [63]	PRF used; not PRGF
Starzyńska et al. (2021) [64]	Advanced platelet-rich fibrin (A-PRF), NOT PRGF
Tatullo et al. (2012) [65]	PRF used, not PRGF
Thakkar et al. (2016) [66]	DFDBA with PRF; not PRGF

PRF—Platelet-rich fibrin, DFDBA—demineralized freeze-dried bone allograft, LPRF—Leucocyte, Platelet-rich fibrin, APRF—advanced platelet-rich fibrin, PRGF—Platelet-rich in growth factors. RCT—Randomized controlled trial, HA—Hydroxyapatite, PRP—platelet-rich plasma, CGF—Concentrated growth factor.

**Table 2:** Details of study type, participants, intervention, outcomes, and conclusion of studies included.

Authors/Year	Study Type	Population	Intervention	Control	Follow-Up Period	Primary Outcomes	Secondary Outcomes	Conclusion
O’Sullivan L. et al./2022 [19].	Prospective Double-blind Randomized Controlled Trial, parallel group design	74 patients (36 PRGF,38 Control) aged 18–40 (28.1 ± 5.8) undergoing impacted mandibular third molar removal	Autologous PRGF application in surgical sockets	Standard surgical procedure without PRGF	3 days and 7 days	Pain (NRS scale), Quality of Life (OHIP-14), Postoperative Symptom Severity (PoSSe)	Mouth opening (MIO), dry socket incidence, socket healing (Landry index), analgesic consumption	Pain scores slightly higher in PRGF group on day 3. No significant differences in OHIP-14 or PoSSe. Secondary outcomes showed no significant differences.
Brazdeikytė V. et al./2021 [24].	prospective, single-center parallel multiple-group randomized Controlled Trial	43 patients (33 female, 10 male) with mandibular molar extraction (mean age 28.6 y) (aged 18–48 years)	PRGF and PRF application for socket regeneration	Hemostatic sponge with gentamicin	1 day and 7 days	Bone regeneration evaluated via CBCT, pain (VAS)	Vertical and diagonal bone loss in alveolus	PRGF had better osteoblastic properties and lower postoperative pain than PRF. Vertical dimensions were preserved in PRGF group but reduced in PRF group.
Stumbras A. et al./2020 [22].	Randomized Controlled parallel-design Trial	60 patients with alveolar ridge preservation	PRGF application vs. xenografts and allografts	Control group without socket preservation	12 weeks	Bone regeneration, ridge width preservation	CBCT measurements	PRGF was equally effective as xenografts/allografts in ridge preservation and bone regeneration.
King E.M. et al./2018 [27].	Single-center, single-blind parallel Randomized Controlled Trial	38 patients (20 male and 18 female) mean age 40.7 ± 17.3, with alveolar osteitis (44 sockets)	PRGF applied in sockets	Alvogyl treatment	3 days, 7 days	Pain, bone coverage, inflammation, halitosis	Quality of life, dysgeusia	PRGF showed faster bone coverage, reduced inflammation, and halitosis but no differences in pain or quality of life.
Anitua E. et al./2015 [28].	Randomized, conventional-treatment, Controlled parallel-design Clinical Trial	60 patients (Median 57 y for PRGF, 53.0 Y control) with mandibular molar extraction 36 subjects PRGF, 24 Control	Autologous PRGF application in extraction sockets	Conventional healing with blood clot	3 days, 7 days, 15 days	Percentage of regenerated socket volume ≥ 75%, bone density	Pain, inflammation, soft tissue healing scores, histological outcomes	PRGF showed significant improvement in socket regeneration, reduced pain, and inflammation, with enhanced soft tissue healing and bone density.
Farina R. et al./2013 [12].	Parallel-arm, open-label, Controlled Clinical Trial	28 patients (mean age-55.2 y, 13 male 15 female).	PRGF application in sockets	Spontaneous healing	4 to 6 weeks, 7 to 10 weeks	Bone volume, tissue mineral content, bone deposition	Histomorphometric markers (CD68+, OCN)	No significant enhancement in early bone deposition with PRGF compared to control.
Mozzati M. et al./2010 [18].	Split-mouth Clinical Trial	60 patients (18–35 y, 22.5 y mean) undergoing bilateral third molar extractions.	Autologous PRGF application in one socket; control with standard care	No PRGF application	2 h and 7 days	Pain, swelling, and healing time	Cytokine production (TNF-α, IL-6)	PRGF reduced pain and swelling and accelerated healing. Cytokine levels were modulated in PRGF-treated sockets.

PRGF—Plasma Rich in Growth Factors, VAS—Visual Analog Scale, OCN—Osteocacin, CBCT—Cone-Beam Computed Tomography, NRS—Numerical rating scale, OHIP—Oral Health Impact Profile.

**Table 3:** Results of the studies included in the systematic review.

Authors	Mineralized Tissue (PRGF Group)	Mineralized Tissue (Control)	Non-Mineralized Tissue (PRGF Group)	Non-Mineralized Tissue (Control)	Healing Index (PRGF Group)	Healing Index (Control)	Pain (PRGF Group)	Pain (Control)	*p*-Value
O’Sullivan L. et al./2022 [19].	NR	NR	NR	NR	Landry 3.6 ± 1.2	Landry 4.0 ± 1.2	NRS 2.7 ± 2.2	NRS 3.2 ± 2.6	*p* > 0.05
Brazdeikytė V. et al./2021 [24].	8.2–10.3 mm	7.5–10.0 mm	NR	NR	NR	NR	VAS 4.4 ± 2.6 *	VAS 5.1 ± 2.8	*p* < 0.05
Stumbras A. et al./2020 [22].	75.5 ± 16.3% *	46.5 ± 15.2%	24.4 ± 16.3% *	53.5 ± 15.2%	NR	NR	NR	NR	*p* < 0.05
King E.M. et al./2018 [27].	NR	NR	Bone coverage faster (PRGF)	Slower coverage (Alvogyl)	Landry 6.7 ± 2.7	Landry 5.6 ± 2.9	VAS 2.0 ± 2.0	VAS 2.4 ± 2.6	*p* > 0.05 pain; Sig inflammation
Anitua E. et al./2015 [28].	450.0 ± 106.7 HU **	318.2 ± 113.0 HU	415.4 ± 140.7 (units) **	274.8 ± 36.0	4.97 ± 0.2 **	3.96 ± 0.6	VAS 0.0	VAS 0.0	*p* = 0.0001
Farina R. et al./2013 [12].	485.8 ± 73.1 mg/cm^3^	538.4 ± 106.6 mg/cm^3^	NR	NR	NR	NR	NR	NR	*p* > 0.05
Mozzati M. et al./2010 [18].	NR	NR	Col I: 12 ± 2.8; Col III: 7 ± 1.8 (units)	Col I: 4 ± 0.9; Col III: 1.9 ± 0.3 (units)	NR	NR	VAS 0.19 cm	VAS 0.49 cm	*p* < 0.05

VAS—Visual Analog Scale, NRS—Numerical rating scale, NR—Not reported, * *p* < 0.05, ** *p* = 0.0001

**Table 4:** The risk of bias assessment.

Study	Randomization Bias	Deviations from Interventions Bias	Missing Data Bias	Outcome Measurement Bias	Reported Results Bias	Overall Risk
L. O’Sullivan et al. [19].	Low	Low	Low	Low	Low	Low
Brazdeikytė V. et al. [24]	Low	Moderate	Low	Moderate	Moderate	Moderate
Stumbras A. et al. [22].	Low	Low	Low	Low	Low	Low
King E.M. et al. [27]	Low	Low	Moderate	Low	Low	Low
Anitua E. et al. [28]	Low	Low	Low	Low	Low	Low
Farina R. et al. [12]	Low	Low	Low	Low	Low	Low
Mozzati M. et al. [18]	Moderate	Low	Moderate	Low	Low	Moderate

## Data Availability

The original contributions presented in this study are included in the article. Further inquiries can be directed to the corresponding author.

## Supplementary Materials

The following supporting information can be downloaded at: https://www.mdpi.com/article/10.3390/jcm15020593/s1, Supplementary Table S1. Heterogenicity assessment. Supplementary File S2. Search Strategies. Supplementary File S3. PRISMA Main Checklist. Supplementary File S4. PRISMA Abstract Checklist. References [68,69,70] are cited in the supplementary materials.

## Abbreviations

PRGF	Plasma Rich in Growth Factors
