# Supplementary material for: Effectiveness of Autologous Plasma Rich in Growth Factors on Healing of Extraction Socket—A Systematic Review
Source: J Clin Med. 2026 Jan 12;15(2):593. doi: 10.3390/jcm15020593 (PMC12842386; doi:10.3390/jcm15020593)
Supplement: Supplementary file 1 [file jcm-15-00593-s001.zip › Supplementary file- 2-Search Stratagies.pdf]

Supplementary file- Boolean Search Strategies for Databases

PubMed (MEDLINE via PubMed)

("Plasma Rich in Growth Factors"[Title/Abstract] OR "PRGF"[Title/Abstract]
OR "platelet rich growth factors"[Title/Abstract]
OR "platelet-rich fibrin"[MeSH Terms] OR "platelet-rich plasma"[MeSH Terms])
AND
("Tooth Extraction"[MeSH Terms] OR extraction[Title/Abstract]
OR "extraction socket"[Title/Abstract]
OR "post-extraction"[Title/Abstract])
AND
(healing[Title/Abstract] OR "socket healing"[Title/Abstract]
OR "bone regeneration"[Title/Abstract] OR "soft tissue healing"[Title/Abstract]
OR pain[Title/Abstract] OR inflammation[Title/Abstract])

OVID MEDLINE

(Plasma Rich in Growth Factors OR PRGF OR platelet rich growth factor*).ti,ab.
OR exp Platelet-Rich Plasma/
OR exp Blood Platelets/

AND

(exp Tooth Extraction/ OR extraction.ti,ab. OR post-extraction.ti,ab.
OR extraction socket.ti,ab.)

AND

(healing.ti,ab. OR bone regeneration.ti,ab. OR soft tissue healing.ti,ab.
OR pain.ti,ab. OR inflammation.ti,ab.)

LIMIT TO (English language AND humans)

EMBASE

('plasma rich in growth factors' OR PRGF OR 'platelet rich growth factor*'
OR 'platelet rich plasma'/exp)

AND

('tooth extraction'/exp OR extraction:ti,ab OR 'extraction socket':ti,ab
OR 'post extraction':ti,ab)

AND

(healing:ti,ab OR 'bone regeneration'/exp OR 'soft tissue healing':ti,ab
OR pain:ti,ab OR inflammation:ti,ab)

AND [english]/lim AND [human]/lim

Cochrane Library

("Plasma Rich in Growth Factors" OR PRGF OR "platelet rich growth factor*"
OR "platelet rich plasma")
AND
("tooth extraction" OR "post-extraction" OR "extraction socket")
AND
(healing OR "bone regeneration" OR "soft tissue healing" OR pain OR inflammation)

SCOPUS

(TITLE-ABS-KEY("plasma rich in growth factors" OR PRGF

OR "platelet rich growth factor*"))
AND
(TITLE-ABS-KEY(extraction OR "extraction socket" OR "post extraction"))
AND
(TITLE-ABS-KEY(healing OR "bone regeneration"
OR "soft tissue healing" OR pain OR inflammation))

Web of Science (WoS)

TS=("plasma rich in growth factors" OR PRGF OR "platelet rich growth factor*")
AND
TS=(extraction OR "post-extraction" OR "extraction socket")
AND
TS=(healing OR "bone regeneration" OR "soft tissue healing" OR pain OR inflammation)

CINAHL

(MH "Platelet Rich Plasma" OR "plasma rich in growth factors" OR PRGF
OR "platelet rich growth factor*")
AND
(MH "Tooth Extraction" OR extraction OR "post extraction" OR "extraction socket")
AND
(healing OR "bone regeneration" OR "soft tissue healing" OR pain OR inflammation)
Limiters: English, Human, Clinical Trial

PsycINFO

("plasma rich in growth factors" OR PRGF OR "platelet rich growth factor*")
AND
("tooth extraction" OR "post extraction" OR "extraction socket")
AND

(pain OR "quality of life" OR inflammation OR healing)

ERIC

("plasma rich in growth factors" OR PRGF)
AND (extraction OR "extraction socket")
AND (healing OR clinical outcomes)
