# Supplementary material for: Effectiveness of Autologous Plasma Rich in Growth Factors on Healing of Extraction Socket—A Systematic Review
Source: J Clin Med. 2026 Jan 12;15(2):593. doi: 10.3390/jcm15020593 (PMC12842386; doi:10.3390/jcm15020593)
Supplement: Supplementary file 1 [file jcm-15-00593-s001.zip › Supplementary Table S1- Heterogenicity assessment.pdf]

Supplementary Table S1:Assessment of Clinical and Methodological Heterogeneity

Rationale for Narrative Synthesis: The significant clinical and methodological heterogeneity observed across the included studies, as detailed below, precluded a meaningful meta-analysis. The variations fundamentally compromise the statistical and clinical homogeneity required to pool results meaningfully.

**Domain of Heterogeneity**	**Assessmen t**	**Examples from Included Studies**	**Impact on Pooling Data**
**1. Patient Population & Indication**	**High**	- **O'Sullivan [19], Mozzati [18]:** Healthy patients undergoing impacted 3rd molar extraction.<br>- **King [26]:** Patients with pre-existing alveolar osteitis (dry socket).<br>- **Stumbras [22]:** Patients requiring alveolar ridge preservation with extraction of tooth in anterior maxilla.<br>- **Anitua [27], Farina [12]:** simple molar extraction, or non molar extraction wider age range.	Populations are not comparable. Healing mechanisms and outcomes for an infected socket (dry socket) vs. a pristine extraction socket are fundamentall y different.
**2. PRGF Preparation Protocol**	**High**	- **Centrifugation:** Variation s in speed, time, and number of steps between studies from Anitua et al. vs. others.<br>- **Activation Method:** Use of calcium chloride may vary, affecting fibrin matrix formation and growth factor release kinetics.	The "intervention" (PRGF) is not uniform. Differences in preparation critically influence biological potency, making it

Domain of Heterogeneity	Assessment	Examples from Included Studies	Impact on Pooling Data
		- **Final Product:** Concentration of platelets and growth factors not standardized or consistently reported.	inappropriate to combine results as if they were the same treatment.
**3. Comparison/Control Group**	**High**	- **O'Sullivan [19]:** Standard healing (blood clot).<br>- **Brazdeikytė [24]:** Hemostatic sponge with gentamicin.<br>- **Stumbras [22]:** Xenografts/Allografts.<br>- **King [26]:** Alvogyl (a dry socket paste).	The "control" intervention varies drastically, from passive healing to active antimicrobial or regenerative treatments. This makes the calculated effect size for PRGF dependent on the specific control used.
**4. Primary Outcome Measures & Tools**	**High**	- **Bone Regeneration:** Measured by CBCT (Stumbras [22], Brazdeikytė [24]), Anitua [27] histomorphometry (Farina [12]), or clinical scoring ().<br>- **Pain:** Measured by VAS (multiple studies) or NRS	Outcomes are measured with different tools and scales that are not directly comparable or convertible

Domain of Heterogeneity	Assessment	Examples from Included Studies	Impact on Pooling Data
		(O'Sullivan [19]).<br>- **Soft Tissue Healing:** Landry index ((Anitua [27]) vs. clinical healing scores O'Sullivan [19]).	for meta-analysis.
**5. Timing of Outcome Assessment (Follow-up)**	**High**	- **Pain/Inflammation:** 1 day, 3 days, 7 days O'Sullivan [19]).<br>- **Early Bone Healing:** 4-10 weeks (Farina [12]).<br>- **Ridge Preservation/Bone Regeneration:** 12 weeks (Stumbras [22]).	The healing process is time-dependent. Measuring bone density at 4 weeks vs. 12 weeks captures different biological stages, making data pooling invalid.
**Quantitative Implication (I^2 Statistic)**	**Not Calculable / Would be High**	Given the profound clinical heterogeneity described above, a statistical test for heterogeneity (e.g., I^2) would be misleading and uninformative. If calculated, it would be exceptionally high ($I^2 > 90\%$), confirming that the observed variance is due to fundamental differences in	A meta-analysis would produce a summary effect that is not representative of any real clinical scenario and would be

Domain of Heterogeneity	Assessment	Examples from Included Studies	Impact on Pooling Data
		study design rather than chance. This violates the core assumption of meta-analysis.	statistically unreliable.

Implication: Meta-analysis not appropriate; descriptive synthesis with GRADE methodology employed

The decision to perform a narrative synthesis was based on the extensive and incommensurable heterogeneity across the domains of **P**opulation, **I**ntervention, **C**omparison, and **O**utcomes (PICO). A meta-analysis was therefore deemed both clinically irrelevant and statistically inappropriate. The narrative synthesis allows for a more nuanced and accurate interpretation of the findings within their specific clinical and methodological contexts.

References for excluded artcles

1. Fok, M. R., Jin, L. Learn, unlearn, and relearn post-extraction alveolar socket healing: Evolving knowledge and practices. *Journal of dentistry*, **2024,** *145,* 104986.
2. Laforgia, A., Inchingolo, A. D., Riccaldo, L., Avantario, P., Buongiorno, S., Malcangi, G., Bordea, I. R., Palermo, A., Inchingolo, F., Inchingolo, A. M., & Dipalma, G. The Use of Platelet-Rich Fibrin (PRF) in the Management of Dry Socket: A Systematic Review. *International journal of molecular sciences*, **2024**, *25*(18), 10069.
3. Al-Maawi, S., Becker, K., Schwarz, F., Sader, R., & Ghanaati, S. Efficacy of platelet-rich fibrin in promoting the healing of extraction sockets: a systematic review. *International journal of implant dentistry*, **2021**, *7*(1), 117.
4. Simon, B. I., Zatcoff, A. L., Kong, J. J., & O'Connell, S. M. Clinical and Histological Comparison of Extraction Socket Healing Following the Use of Autologous Platelet-Rich Fibrin Matrix (PRFM) to Ridge Preservation Procedures Employing Demineralized Freeze Dried Bone Allograft Material and Membrane. *The open dentistry journal*, **2009,** *3*, 92–99.
5. Ucer, C., & Khan, R. S. Alveolar Ridge Preservation with Autologous Platelet-Rich Fibrin (PRF): Case Reports and the Rationale. *Dentistry journal*, **2023**, *11*(10), 244.
6. Mozzati, M., Tumedei, M., Gallesio, G., Menicucci, G., Manzella, C., Testori, T., & Fabbro, M. D. Healing of Alveolar Sockets Treated with Concentrated Growth Factors: A Split-Mouth Study. *Materials (Basel, Switzerland)*, **2022**, *15*(14), 4859.
7. Hatakeyama, I., Marukawa, E., Takahashi, Y., & Omura, K. Effects of platelet-poor plasma, platelet-rich plasma, and platelet-rich fibrin on healing of extraction sockets with buccal dehiscence in dogs. *Tissue engineering. Part A*, **2014**, *20*(3-4), 874–882.
8. Kamal, A., Salman, B., Abdul Razak, N. H., Qabbani, A. A., & Samsudin, A. R. The Efficacy of Concentrated Growth Factor in the Healing of Alveolar Osteitis: A Clinical Study. *International journal of dentistry*, *2020*, **2020**, 9038629.
9. Dutta, S. R., Passi, D., Singh, P., Sharma, S., Singh, M., & Srivastava, D. A randomized comparative prospective study of platelet-rich plasma, platelet-rich fibrin, and hydroxyapatite as a graft material for mandibular third molar extraction socket healing. *National journal of maxillofacial surgery*, **2016**, *7*(1), 45–51.
10. Guo, X., Lu, H., Liu, C., Zhang, Y., & Bi, L. Effects of Super-Activated Platelet Lysate on Early Healing of Tooth Extraction Sockets in Rats. *Drug design, development and therapy*, *16*, **2022,** 2213–2227.
11. Mogharehabed, A., Birang, R., Torabinia, N., Nasiri, S., & Behfarnia, P. Socket preservation using demineralized freezed dried bone allograft with and without plasma rich in growth factor: A canine study. *Dental research journal*, **2014,** *11*(4), 460–468.
12. Dohan Ehrenfest D.M., Del Corso M., Inchingolo F., Sammartino G., Charrier J.-B. Platelet-Rich Plasma (PRP) and Platelet-Rich Fibrin (PRF) in Human Cell Cultures: Growth Factor Release and Contradictory Results. Oral Surg. Oral Med. Oral Pathol. Oral Radiol. Endod. 2010;110:418–421; author reply 421–422.
13. Starzyńska A., Kaczoruk-Wieremczuk M., Lopez M.A., Passarelli P.C., Adamska P. The Growth Factors in Advanced Platelet-Rich Fibrin (A-PRF) Reduce Postoperative Complications after Mandibular Third Molar Odontectomy. Int. J. Environ. Res. Public Health. 2021;18:13343.
14. Tatullo M., Marrelli M., Cassetta M., Pacifici A., Stefanelli L.V., Scacco S., Dipalma G., Pacifici L., Inchingolo F. Platelet Rich Fibrin (P.R.F.) in Reconstructive Surgery of Atrophied Maxillary Bones: Clinical and Histological Evaluations. Int. J. Med. Sci. 2012;9:872–880.

15. Asutay F., Yolcu Ü., Geçör O., Acar A., Öztürk S., Malkoç S. An Evaluation of Effects of Platelet-Rich-Fibrin on Postoperative Morbidities after Lower Third Molar Surgery. Niger. J. Clin. Pract. 2017;20:1531–1536.
16. Fang D., Li D., Li C., Yang W., Xiao F., Long Z. Efficacy and Safety of Concentrated Growth Factor Fibrin on the Extraction of Mandibular Third Molars: A Prospective, Randomized, Double-Blind Controlled Clinical Study. J. Oral Maxillofac. Surg. Off. J. Am. Assoc. Oral Maxillofac. Surg. 2022;80:700–708.
17. Vitenson J., Starch-Jensen T., Bruun N.H., Larsen M.K. The Use of Advanced Platelet-Rich Fibrin after Surgical Removal of Mandibular Third Molars: A Systematic Review and Meta-Analysis. Int. J. Oral Maxillofac. Surg. 2022;51:962–974.
18. Borsani E., Bonazza V., Buffoli B., Nocini P.F., Albanese M., Zotti F., Inchingolo F., Rezzani R., Rodella L.F. Beneficial Effects of Concentrated Growth Factors and Resveratrol on Human Osteoblasts In Vitro Treated with Bisphosphonates. BioMed Res. Int. 2018;2018:4597321.
19. Dohan Ehrenfest D.M., Bielecki T., Jimbo R., Barbe G., Del Corso M., Inchingolo F., Sammartino G. Do the Fibrin Architecture and Leukocyte Content Influence the Growth Factor Release of Platelet Concentrates? An Evidence-Based Answer Comparing a Pure Platelet-Rich Plasma (P-PRP) Gel and a Leukocyte- and Platelet-Rich Fibrin (L-PRF) Curr. Pharm. Biotechnol. 2012;13:1145–1152.
20. Semkin V.A., Gurin A.N., Vitrenko D.V., Levchenko D.D. [Prevention of inflammatory complications after surgical extraction of mandibular third molars] Stomatologiia. 2022;101:38–43.
21. Santos Pereira V.B., da Silva Barbirato D., do Lago C.A.P., do Egito Vasconcelos B.C. The Effect of Advanced Platelet-Rich Fibrin in Tissue Regeneration in Reconstructive and Graft Surgery: Systematic Review. J. Craniofac. Surg. 2023;34:1217–1221.
22. Anwandter A, Bohmann S, Nally M, Castro AB, Quirynen M, Pinto N. Dimensional changes of the post extraction alveolar ridge, preserved with Leukocyte- and Platelet Rich Fibrin: a clinical pilot study. J Dent. 2016;52:23–9.
23. Schär MO, Diaz-Romero J, Kohl S, Zumstein MA, Nesic D. Platelet-rich con centrates differentially release growth factors and induce cell migration in vitro. Clin Orthop Relat Res. 2015;473(5):1635–43.
24. Kubesch A, Barbeck M, Al-Maawi S, Orlowska A, Booms PF, Sader RA, et al. A low-speed centrifugation concept leads to cell accumulation and vascularization of solid platelet-rich fibrin: an experimental study in vivo. Platelets. 2018; pp. 1–12.
25. Kobayashi E, Flückiger L, Fujioka-Kobayashi M, Sawada K, Sculean A, Schaller B, et al. Comparative release of growth factors from PRP, PRF, and advanced-PRF. Clin Oral Investig. 2016; pp. 1–8.
26. Anitua E, Sánchez M, Orive G, Andía I. The potential impact of the prepa ration rich in growth factors (PRGF) in different medical fields. Biomateri als. 2007;28(31):4551–60
27. Anitua E, Andia I, Sanchez M. PRGF plasma rich growth factors. Dent dialogue. 2004; pp. 1–9.
28. Choukroun J, Diss A, Simonpieri A, Girard M-O, Schoeffler C, Dohan SL, et al. Platelet-rich fibrin (PRF): a second-generation platelet concentrate. Part IV: clinical effects on tissue healing. Oral Surg Oral Med Oral Pathol Oral Radiol Endodontol. 2006;101(3):56–60.
29. Ghanaati S, Herrera-Vizcaino C, Al-Maawi S, Lorenz J, Miron RJ, Nelson K, et al. Fifteen years of platelet rich fibrin (PRF) in dentistry and oromaxil lofacial surgery: how high is the level of scientific evidence? J Oral Implantol. 2018;44:471.
30. Clark D, Rajendran Y, Paydar S, Ho S, Cox D, Ryder M, et al. Advanced platelet-rich fibrin and freeze-dried bone allograft for ridge preservation: a randomized controlled clinical trial. J Periodontol. 2018;89(4):379–87.

31. Giudice A, Esposito M, Bennardo F, Brancaccio Y, Buti J, Fortunato L. Dental extractions for patients on oral antiplatelet: a within-person randomised controlled trial comparing haemostatic plugs, advanced platelet-rich fibrin (A-PRF+) plugs, leukocyte- and platelet-rich fibrin (L-PRF) plugs and suturing alone; 2019.
32. Castro AB, Van Dessel J, Temmerman A, Jacobs R, Quirynen M. Effect of different platelet-rich fibrin matrices for ridge preservation in multiple tooth extractions: a split-mouth randomized controlled clinical trial. J Clin Periodontol. 2021;48:984.
33. Ustaoğlu G, Göller Bulut D, Gümüş K. Evaluation of different platelet-rich concentrates effects on early soft tissue healing and socket preservation after tooth extraction. J Stomatol Oral Maxillofac Surg. 2019.

34. Girish Kumar N, Chaudhary R, Kumar I, Arora SS, Kumar N, Singh H. To assess the efficacy of socket plug technique using platelet rich fibrin with or without the use of bone substitute in alveolar ridge preserva tion: a prospective randomised controlled study. Oral Maxillofac Surg. 2018;22(2):135–42.

35. Alzahrani AA, Murriky A, Shafik S. Influence of platelet rich fibrin on post extraction socket healing: a clinical and radiographic study. Saudi Dent J. 2017;29(4):149–55.

36. Hauser F, Gaydarov N, Badoud I, Vazquez L, Bernard JP, Ammann P. Clinical and histological evaluation of postextraction platelet-rich fibrin socket filling: a prospective randomized controlled study. Implant Dent. 2013;22(3):295–303.

37. Srinivas B, Das P, Rana MM, Qureshi AQ, Vaidya KC, Raziuddin SJA. Wound healing and bone regeneration in postextraction sockets with and with out platelet-rich fibrin. Ann Maxillofac Surg. 2018;8(1):28–34.

38. Thakkar DJ, Deshpande NC, Dave DH, Narayankar SD. A comparative evaluation of extraction socket preservation with demineralized freeze dried bone allograft alone and along with platelet-rich fibrin: a clinical and radiographic study. Contemp Clin Dent. 2016;7(3):371–6.

39. Ahmed N, Gopalakrishna V, Shetty A, Nagraj V, Imran M, Kumar P. Efficacy of PRF vs PRF + biodegradable collagen plug in post-extraction preserva tion of socket. J Contemp Dent Pract. 2019;20(11):1323–8.
